# *N*-(4-Chloro­phen­yl)-4-nitro­benzamide

**DOI:** 10.1107/S1600536812036082

**Published:** 2012-08-25

**Authors:** Ghulam Waris, Humaira Masood Siddiqi, Ulrich Flörke, M.Saeed Butt, Rizwan Hussain

**Affiliations:** a Department of Chemistry, Quaid - I- Azam University, Islamabad 45320, Pakistan; b Universität Paderborn, Warburgerstrasse 100, D-33098 Paderborn, Germany; c NESCOM, PO Box 2216, Islamabad, Pakistan

## Abstract

The title compound, C_13_H_9_ClN_2_O_3_, is almost planar, showing a dihedral angle of 4.63 (6)° between the aromatic ring planes. The nitro group also lies in the plane, the C—C—N—O torsion angle being 6.7 (2)°. There is an intamolecular C—H⋯O hydrogen bond. The crystal structure features N—H⋯O(nitro) hydrogen bonds that link the mol­ecules into zigzag chains extending along [010].

## Related literature

For background information on aromatic polyimides, see: Yang *et al.* (1999 ▶); More *et al.* (2010 ▶); Litvinov *et al.*, (2010 ▶); Sheng *et al.* (2009 ▶); Choi *et al.* (1992 ▶); Hsiao & Lin (2004 ▶); Li *et al.* (2007 ▶); Liaw *et al.* (2005 ▶). For related structures, see Saeed *et al.* (2011 ▶); Wardell *et al.* (2006 ▶).
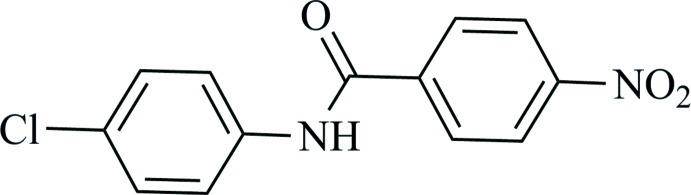


## Experimental

### 

#### Crystal data

C_13_H_9_ClN_2_O_3_
*M* *_r_* = 276.67
Monoclinic, 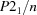

*a* = 9.6019 (7) Å
*b* = 13.0688 (10) Å
*c* = 9.6412 (7) Å
β = 103.853 (1)°
*V* = 1174.64 (15) Å^3^
*Z* = 4
Mo *K*α radiation
μ = 0.33 mm^−1^
*T* = 130 K
0.49 × 0.20 × 0.18 mm

#### Data collection

Bruker SMART APEX diffractometer
Absorption correction: multi-scan (*SADABS*; Sheldrick, 2004 ▶) *T*_min_ = 0.855, *T*_max_ = 0.943
10822 measured reflections
2808 independent reflections
2557 reflections with *I* > 2σ(*I*)
*R*_int_ = 0.019

#### Refinement

*R*[*F* ^2^ > 2σ(*F* ^2^)] = 0.042
*wR*(*F* ^2^) = 0.116
*S* = 1.08
2808 reflections
172 parameters
H-atom parameters constrained
Δρ_max_ = 0.76 e Å^−3^
Δρ_min_ = −0.26 e Å^−3^

### 

Data collection: *SMART* (Bruker, 2002 ▶); cell refinement: *SAINT* (Bruker, 2002 ▶); data reduction: *SAINT*; program(s) used to solve structure: *SHELXTL* (Sheldrick, 2008 ▶); program(s) used to refine structure: *SHELXTL*; molecular graphics: *SHELXTL*; software used to prepare material for publication: *SHELXTL* and local programs.

## Supplementary Material

Crystal structure: contains datablock(s) I, global. DOI: 10.1107/S1600536812036082/bt6822sup1.cif

Structure factors: contains datablock(s) I. DOI: 10.1107/S1600536812036082/bt6822Isup2.hkl

Supplementary material file. DOI: 10.1107/S1600536812036082/bt6822Isup3.cml

Additional supplementary materials:  crystallographic information; 3D view; checkCIF report

## Figures and Tables

**Table 1:** Hydrogen-bond geometry (Å, °)

*D*—H⋯*A*	*D*—H	H⋯*A*	*D*⋯*A*	*D*—H⋯*A*
C13—H13*A*⋯O1	0.95	2.26	2.859 (2)	120
N1—H1*A*⋯O3^i^	0.88	2.29	3.1312 (17)	159

Symmetry code: (i) 
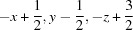
.

**Fig. 1.**
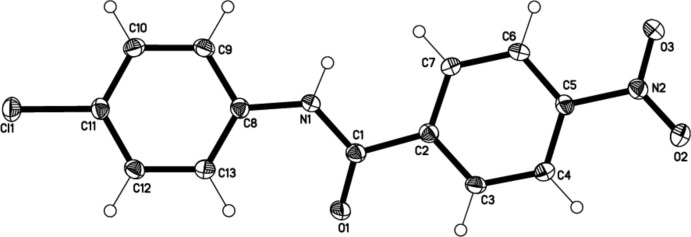
Molecular structure of the title compound. Displacement ellipsoids are drawn at the 50% probability level.

**Fig. 2.**
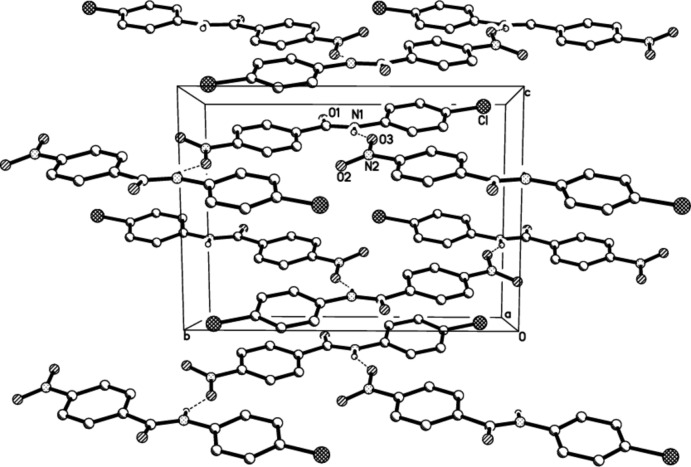
Crystal packing viewed along [100] with hydrogen bonding pattern indicated as dashed lines. H-atoms not involved are omitted.

## supplementary crystallographic information

### Comment

Aromatic polyimides are distinguished as high performance polymers owing to excellent thermal, mechanical, and chemical properties (Yang *et al.*, 1999, More *et al.*, 2010). They are not only used as beneficial substitutes for metals or ceramics in presently used goods but also as new materials in novel technological applications (Litvinov *et al.*, 2010). Nevertheless, infusibility and insolubility are some of the shortcomings due to the highly regular and rigid polymer backbones and the formation of intermolecular hydrogen bonding, causing deterioration in processability and applications (Sheng *et al.*, 2009, Choi *et al.*, 1992). In order to improve upon these drawbacks, recent research has aimed at improving their processability and solubility without an intense loss in the chemical, thermal, and mechanical properties. For this, improvement of solubility is targeted through diminishing the cohesive energy by lowering the interchain interactions. To achieve this, designing and synthesizing new diamines or dicarboxylic acids is proposed to produce a great variety of soluble and processable polyimides (Hsiao *et al.*, 2004). Incorporating substituted pendant groups which reduce dense chain packing and interchain interactions increases the solubility of resulting polyimides (Liaw *et al.*, 2005, Li *et al.*, 2007). As part of our enduring interest in solubility of aromatic polyimides by structural modification, we are reporting a chloro substituted pendant group having inbuilt amide functionality, which enhances the solubility of polyimides without worsening the inherent properties of polyimides. The molecular structure of the title compound (Figure 1) is closely related to that of the bromo- (Saeed *et al.*, 2011) and iodo-compound (Wardell *et al.*, 2006). The two aromatic rings are almost coplanar with a dihedral angle of 4.63 (6)°, and the nitro group is also coplanar, the associated C4–C5–N2–O2 torsion angle is 6.7 (2)°. The molecular conformation is stabilized by a rather strong intramolecular C13–H···O1 bond. Crystal packing shows a strong intermolecular N1–H···O3(-*x* + 0.5, *y* - 0.5, -*z* + 1.5) hydrogen interaction with H···O3 2.29 Å and N–H···O 159.1° that links molecules into endless zigzag chains extended along the *b* axis (Figure 2).

### Experimental

All the chemicals were of analytical grade and no further purification was carried out before their usage. 1.275 g (0.01 mole) of 4-chloroaniline, 25 ml dichloromethane and 1.39 ml of triethylamine were charged in 100 ml, three-necked, round-bottomed flask fitted with a condenser, a nitrogen inlet tube, a thermometer and a magnetic stirrer. The mixture was stirred at 273-278K for 30 minutes. A solution of 1.85 g (0.01mole) of 4-nitrobenzoyl chloride in 25 ml dichloromethane was added dropwise and stirring was continued for further 45 minutes under same conditions. The temperature was then raised to room temperature along with stirring for further 30 minutes. Product was precipitated by pouring the flask content into water. The product was filtered, washed with 5% NaOH solution, further washing with hot water was carried out and solid product was dried overnight under vacuum at 343K. The product was recrystallized from an ethanol-tetrahydrofuran(1:1)

### Refinement

Hydrogen atoms were clearly derived from difference Fourier maps and then refined at idealized positions riding on the carbon or nitrogen atoms with isotropic displacement parameters *U*_iso_(H) = 1.2U(C/N_eq_) and N—H 0.88 / C—H 0.95 Å.

### Crystal data

**Table:**

C_13_H_9_ClN_2_O_3_	*F*(000) = 568
*M**_r_* = 276.67	*D*_x_ = 1.564 Mg m^−^^3^
Monoclinic, *P*2_1_/*n*	Melting point: 141 K
Hall symbol: -P 2yn	Mo *K*α radiation, λ = 0.71073 Å
*a* = 9.6019 (7) Å	Cell parameters from 5166 reflections
*b* = 13.0688 (10) Å	θ = 2.7–28.3°
*c* = 9.6412 (7) Å	µ = 0.33 mm^−^^1^
β = 103.853 (1)°	*T* = 130 K
*V* = 1174.64 (15) Å^3^	Prism, yellow
*Z* = 4	0.49 × 0.20 × 0.18 mm

### Data collection

**Table:**

Bruker SMART APEX diffractometer	2808 independent reflections
Radiation source: sealed tube	2557 reflections with *I* > 2σ(*I*)
Graphite monochromator	*R*_int_ = 0.019
φ and ω scans	θ_max_ = 27.9°, θ_min_ = 2.7°
Absorption correction: multi-scan (*SADABS*; Sheldrick, 2004)	*h* = −11→12
*T*_min_ = 0.855, *T*_max_ = 0.943	*k* = −16→17
10822 measured reflections	*l* = −12→12

### Refinement

**Table:**

Refinement on *F*^2^	Primary atom site location: structure-invariant direct methods
Least-squares matrix: full	Secondary atom site location: difference Fourier map
*R*[*F*^2^ > 2σ(*F*^2^)] = 0.042	Hydrogen site location: difference Fourier map
*wR*(*F*^2^) = 0.116	H-atom parameters constrained
*S* = 1.08	*w* = 1/[σ^2^(*F*_o_^2^) + (0.0652*P*)^2^ + 0.6505*P*] where *P* = (*F*_o_^2^ + 2*F*_c_^2^)/3
2808 reflections	(Δ/σ)_max_ < 0.001
172 parameters	Δρ_max_ = 0.76 e Å^−^^3^
0 restraints	Δρ_min_ = −0.26 e Å^−^^3^

### Special details

**Table:**

Geometry. All e.s.d.'s (except the e.s.d. in the dihedral angle between two l.s. planes) are estimated using the full covariance matrix. The cell e.s.d.'s are taken into account individually in the estimation of e.s.d.'s in distances, angles and torsion angles; correlations between e.s.d.'s in cell parameters are only used when they are defined by crystal symmetry. An approximate (isotropic) treatment of cell e.s.d.'s is used for estimating e.s.d.'s involving l.s. planes.
Refinement. Refinement of *F*^2^ against ALL reflections. The weighted *R*-factor *wR* and goodness of fit *S* are based on *F*^2^, conventional *R*-factors *R* are based on *F*, with *F* set to zero for negative *F*^2^. The threshold expression of *F*^2^ > σ(*F*^2^) is used only for calculating *R*-factors(gt) *etc*. and is not relevant to the choice of reflections for refinement. *R*-factors based on *F*^2^ are statistically about twice as large as those based on *F*, and *R*- factors based on ALL data will be even larger.

### Fractional atomic coordinates and isotropic or equivalent isotropic displacement parameters (Å^2^)

**Table:**

	*x*	*y*	*z*	*U*_iso_*/*U*_eq_	
Cl1	0.86234 (4)	0.07922 (3)	0.98180 (4)	0.02702 (14)	
O1	0.81457 (12)	0.60046 (9)	0.92017 (14)	0.0290 (3)	
O2	0.38514 (13)	1.02953 (9)	0.81384 (14)	0.0317 (3)	
O3	0.20735 (12)	0.93858 (9)	0.69700 (13)	0.0268 (3)	
N1	0.61843 (14)	0.49653 (10)	0.87249 (14)	0.0212 (3)	
H1A	0.5241	0.4972	0.8458	0.025*	
N2	0.33274 (14)	0.94804 (11)	0.76579 (15)	0.0216 (3)	
C1	0.68462 (17)	0.58903 (12)	0.88325 (17)	0.0208 (3)	
C2	0.58628 (16)	0.68089 (12)	0.84829 (16)	0.0193 (3)	
C3	0.64838 (16)	0.77663 (12)	0.88474 (17)	0.0210 (3)	
H3A	0.7479	0.7812	0.9287	0.025*	
C4	0.56698 (17)	0.86526 (12)	0.85776 (17)	0.0215 (3)	
H4A	0.6090	0.9305	0.8833	0.026*	
C5	0.42207 (16)	0.85577 (11)	0.79222 (16)	0.0193 (3)	
C6	0.35741 (16)	0.76187 (13)	0.75241 (17)	0.0217 (3)	
H6A	0.2584	0.7578	0.7064	0.026*	
C7	0.44026 (17)	0.67427 (12)	0.78121 (17)	0.0224 (3)	
H7A	0.3977	0.6092	0.7553	0.027*	
C8	0.68397 (16)	0.39913 (12)	0.89933 (16)	0.0198 (3)	
C9	0.59677 (17)	0.31396 (12)	0.85279 (17)	0.0220 (3)	
H9A	0.4996	0.3238	0.8032	0.026*	
C10	0.65021 (17)	0.21567 (13)	0.87802 (17)	0.0225 (3)	
H10A	0.5907	0.1580	0.8466	0.027*	
C11	0.79284 (17)	0.20303 (12)	0.95039 (17)	0.0203 (3)	
C12	0.88114 (16)	0.28597 (13)	0.99628 (17)	0.0213 (3)	
H12A	0.9784	0.2756	1.0452	0.026*	
C13	0.82716 (17)	0.38472 (13)	0.97057 (17)	0.0215 (3)	
H13A	0.8875	0.4420	1.0014	0.026*	

### Atomic displacement parameters (Å^2^)

**Table:**

	*U*^11^	*U*^22^	*U*^33^	*U*^12^	*U*^13^	*U*^23^
Cl1	0.0277 (2)	0.0195 (2)	0.0341 (2)	0.00400 (14)	0.00786 (16)	0.00411 (15)
O1	0.0156 (5)	0.0224 (6)	0.0469 (7)	−0.0015 (4)	0.0033 (5)	0.0064 (5)
O2	0.0278 (6)	0.0180 (6)	0.0468 (8)	−0.0001 (5)	0.0041 (5)	−0.0024 (5)
O3	0.0182 (5)	0.0259 (6)	0.0344 (6)	0.0029 (4)	0.0029 (5)	−0.0003 (5)
N1	0.0142 (6)	0.0192 (6)	0.0288 (7)	0.0001 (5)	0.0023 (5)	0.0008 (5)
N2	0.0194 (6)	0.0216 (7)	0.0248 (6)	0.0008 (5)	0.0069 (5)	0.0004 (5)
C1	0.0177 (7)	0.0216 (8)	0.0232 (7)	−0.0007 (6)	0.0051 (6)	0.0026 (6)
C2	0.0177 (7)	0.0203 (7)	0.0202 (7)	−0.0005 (5)	0.0053 (5)	0.0019 (5)
C3	0.0153 (6)	0.0227 (8)	0.0238 (7)	−0.0022 (6)	0.0024 (6)	0.0009 (6)
C4	0.0199 (7)	0.0195 (7)	0.0247 (7)	−0.0033 (6)	0.0046 (6)	−0.0005 (6)
C5	0.0183 (7)	0.0195 (7)	0.0208 (7)	0.0021 (6)	0.0063 (5)	0.0006 (5)
C6	0.0146 (6)	0.0243 (8)	0.0248 (8)	−0.0014 (6)	0.0023 (5)	−0.0006 (6)
C7	0.0188 (7)	0.0187 (7)	0.0286 (8)	−0.0029 (6)	0.0033 (6)	−0.0014 (6)
C8	0.0191 (7)	0.0194 (7)	0.0218 (7)	0.0014 (6)	0.0067 (6)	0.0012 (6)
C9	0.0158 (7)	0.0249 (8)	0.0242 (7)	0.0002 (6)	0.0026 (6)	−0.0010 (6)
C10	0.0195 (7)	0.0218 (8)	0.0267 (8)	−0.0037 (6)	0.0063 (6)	−0.0026 (6)
C11	0.0203 (7)	0.0183 (7)	0.0237 (7)	0.0027 (6)	0.0083 (6)	0.0027 (6)
C12	0.0159 (7)	0.0239 (8)	0.0239 (7)	0.0012 (6)	0.0042 (6)	0.0024 (6)
C13	0.0181 (7)	0.0215 (7)	0.0247 (7)	−0.0009 (6)	0.0048 (6)	0.0001 (6)

### Geometric parameters (Å, º)

**Table:**

Cl1—C11	1.7488 (16)	C5—C6	1.387 (2)
O1—C1	1.222 (2)	C6—C7	1.384 (2)
O2—N2	1.2207 (19)	C6—H6A	0.9500
O3—N2	1.2339 (17)	C7—H7A	0.9500
N1—C1	1.358 (2)	C8—C13	1.395 (2)
N1—C8	1.4161 (19)	C8—C9	1.400 (2)
N1—H1A	0.8800	C9—C10	1.383 (2)
N2—C5	1.466 (2)	C9—H9A	0.9500
C1—C2	1.515 (2)	C10—C11	1.390 (2)
C2—C3	1.394 (2)	C10—H10A	0.9500
C2—C7	1.399 (2)	C11—C12	1.382 (2)
C3—C4	1.387 (2)	C12—C13	1.391 (2)
C3—H3A	0.9500	C12—H12A	0.9500
C4—C5	1.389 (2)	C13—H13A	0.9500
C4—H4A	0.9500		
			
C1—N1—C8	127.36 (13)	C5—C6—H6A	120.7
C1—N1—H1A	116.3	C6—C7—C2	120.39 (14)
C8—N1—H1A	116.3	C6—C7—H7A	119.8
O2—N2—O3	123.50 (14)	C2—C7—H7A	119.8
O2—N2—C5	118.73 (13)	C13—C8—C9	119.58 (14)
O3—N2—C5	117.76 (13)	C13—C8—N1	123.64 (14)
O1—C1—N1	123.89 (14)	C9—C8—N1	116.77 (13)
O1—C1—C2	120.44 (14)	C10—C9—C8	120.91 (14)
N1—C1—C2	115.67 (13)	C10—C9—H9A	119.5
C3—C2—C7	119.53 (14)	C8—C9—H9A	119.5
C3—C2—C1	116.68 (13)	C9—C10—C11	118.56 (14)
C7—C2—C1	123.79 (14)	C9—C10—H10A	120.7
C4—C3—C2	120.96 (14)	C11—C10—H10A	120.7
C4—C3—H3A	119.5	C12—C11—C10	121.51 (14)
C2—C3—H3A	119.5	C12—C11—Cl1	119.40 (12)
C3—C4—C5	117.96 (14)	C10—C11—Cl1	119.09 (12)
C3—C4—H4A	121.0	C11—C12—C13	119.80 (14)
C5—C4—H4A	121.0	C11—C12—H12A	120.1
C6—C5—C4	122.55 (14)	C13—C12—H12A	120.1
C6—C5—N2	118.36 (13)	C12—C13—C8	119.63 (14)
C4—C5—N2	119.09 (14)	C12—C13—H13A	120.2
C7—C6—C5	118.60 (14)	C8—C13—H13A	120.2
C7—C6—H6A	120.7		
			
C8—N1—C1—O1	−0.7 (3)	N2—C5—C6—C7	−177.98 (14)
C8—N1—C1—C2	−179.73 (14)	C5—C6—C7—C2	−0.4 (2)
O1—C1—C2—C3	−11.2 (2)	C3—C2—C7—C6	−0.7 (2)
N1—C1—C2—C3	167.93 (14)	C1—C2—C7—C6	−179.81 (15)
O1—C1—C2—C7	167.99 (16)	C1—N1—C8—C13	14.2 (3)
N1—C1—C2—C7	−12.9 (2)	C1—N1—C8—C9	−167.02 (15)
C7—C2—C3—C4	1.2 (2)	C13—C8—C9—C10	0.8 (2)
C1—C2—C3—C4	−179.64 (14)	N1—C8—C9—C10	−178.06 (14)
C2—C3—C4—C5	−0.6 (2)	C8—C9—C10—C11	−0.2 (2)
C3—C4—C5—C6	−0.5 (2)	C9—C10—C11—C12	−0.3 (2)
C3—C4—C5—N2	178.45 (14)	C9—C10—C11—Cl1	−179.48 (12)
O2—N2—C5—C6	172.37 (15)	C10—C11—C12—C13	0.2 (2)
O3—N2—C5—C6	−6.8 (2)	Cl1—C11—C12—C13	179.39 (12)
O2—N2—C5—C4	−6.7 (2)	C11—C12—C13—C8	0.4 (2)
O3—N2—C5—C4	174.13 (14)	C9—C8—C13—C12	−0.9 (2)
C4—C5—C6—C7	1.0 (2)	N1—C8—C13—C12	177.89 (14)

### Hydrogen-bond geometry (Å, º)

**Table:**

*D*—H···*A*	*D*—H	H···*A*	*D*···*A*	*D*—H···*A*
C13—H13*A*···O1	0.95	2.26	2.859 (2)	120
N1—H1*A*···O3^i^	0.88	2.29	3.1312 (17)	159

Symmetry code: (i) −*x*+1/2, *y*−1/2, −*z*+3/2.
